# Amide proton transfer imaging reveals cerebral metabolic alterations associated with cognitive impairment in type 2 diabetes mellitus

**DOI:** 10.1186/s12967-025-07083-0

**Published:** 2025-10-07

**Authors:** Hongjun Jiang, Shuncheng Yu, Langxuan Yu, Wei Du, Chang Yuan, Jiajun Cao, Qingwei Song, Tieli Liu, Yanwei Miao, Weiwei Wang

**Affiliations:** 1 Radiology Department, the First Affiliated Hospital of Dalian Medical University, No.222 Zhongshan Road, Dalian, 116011 Liaoning Province China; 2 College of Medical Imaging, Dalian Medical University, No. 9 West Section of Lvshun South Road, Dalian, 116044 Liaoning Province China

**Keywords:** Amide proton transfer, Type 2 diabetes mellitus, Cognitive dysfunction, Cerebral metabolic changes, MRI

## Abstract

**Background:**

Amide proton transfer (APT) imaging indirectly reflects tissue metabolic changes by detecting variations in the concentration of mobile amide protons and tissue pH. Type 2 diabetes mellitus (T2DM) is often accompanied by cognitive dysfunction and diabetic encephalopathy, both of which pose serious threat to human health and quality of life. This study aimed to evaluate the potential of APT imaging as a novel biomarker for detecting cerebral metabolic alterations and to investigate its associations with cognitive impairment in patients with T2DM.

**Methods:**

This study included 32 T2DM patients, comprising 16 with mild cognitive impairment (MCI) and 16 with normal cognition (NC), and 26 healthy controls. Clinical data and cognitive assessments were collected within one week of MRI acquisition. Imaging markers of cerebral small vessel disease (CSVD) were evaluated using AI-assisted tools. APT values were measured in predefined brain regions, including the hippocampus (hipp), temporal white matter (TWM), temporal gray matter (TGM), occipital white matter (OWM), occipital gray matter (OGM), and cerebral peduncles (CPs) using 3D Slicer software. Group differences were analyzed with one-way ANOVA followed by Bonferroni-corrected post hoc tests (or Kruskal–Wallis test with Bonferroni correction for non-parametric data). Partial correlations (Bonferroni-corrected) assessed the links between APT values and cognitive scores, as well as between APT values and CSVD imaging markers.

**Results:**

The APT values of the left temporal white matter (TWM) and the right temporal gray matter (TGM) were significantly different among the three groups. Among them, the APT values of T2DM-MCI group were significantly lower. In T2DM patients, partial correlation analysis showed that the APT values of the left TWM was positively correlated with MMSE attention and calculation score, MoCA attention score, and the number of lacunar infarcts (LI), and negatively correlated with the severity of white matter hyperintensities (WMH). The APT values of right TGM was positively correlated with MoCA total scores, MoCA visuospatial scores and MoCA delayed recall scores.

**Conclusion:**

T2DM patients with mild cognitive impairment exhibited significantly lower APT values in the left temporal white matter and right temporal gray matter. These lower APT values were strongly associated with poorer cognitive performance and more severe CSVD. APT imaging may serve as a sensitive, noninvasive biomarker for detecting cerebral metabolic deterioration underlying diabetic cognitive decline.

**Supplementary Information:**

The online version contains supplementary material available at 10.1186/s12967-025-07083-0.

## Background

Type 2 diabetes mellitus (T2DM) is a metabolic disorder characterized by progressive pancreatic β-cell dysfunction and insulin resistance, leading to relative insulin deficiency and chronic hyperglycemia [1, 2]. According to the 10th edition of the IDF Diabetes Atlas (2021), the number of individuals with diabetes in China exceeds 140 million, the highest in the world [2]. As the disease progresses, T2DM is associated with chronic complications affecting the vasculature, kidneys, retina, and nervous system [3]. Notably, accumulating evidence indicates that T2DM is associated with an approximately 1.5-fold higher risk of cognitive impairment and dementia compared with healthy individuals [4]. This elevated risk may be partly attributable to atherosclerotic cerebrovascular disease and neurodegeneration [5]. In addition, cerebral small vessel disease (CSVD) shown by conventional MRI is associated with T2DM, and T2DM is an independent risk factor for some CSVD phenotypes, indicating a close relationship between them [6, 7]. Mild cognitive impairment (MCI) represents a transitional stage between normal cognition and dementia, defined by objective cognitive decline without loss of independent daily functioning [8]. The early identification of MCI is mainly based on the presence of subjective cognitive decline, and therefore the neuropsychological assessment of cognitive function serves as a primary clinical tool for its evaluation [9]. While clinical neurocognitive scales like the Montreal Cognitive Assessment (MoCA) and Mini-Mental State Examination (MMSE) are used to screen for cognitive deficits, their results may be influenced by participant-related subjective factors or ceiling effects. Therefore, it is important to integrate these assessments with objective neuroimaging measures such as MRI to improve early detection of diabetic cognitive impairment [10].

Chemical exchange saturation transfer (CEST) imaging is a novel non-contrast MRI technique that detects proton exchange between certain molecular groups and free water. By applying low-power radiofrequency saturation pulses targeting exchangeable protons, CEST amplifies subtle signal changes from proton exchange, enabling quantification of tissue microenvironment parameters such as pH and protein content [11]. Amide proton transfer (APT) imaging, the most widely studied CEST subtype, specifically probes amide proton exchange in endogenous proteins and peptides, serving as a surrogate marker of cellular protein content and metabolism [12]. APT imaging has been successfully applied in various clinical contexts, for example, in the diagnosis and grading of brain tumors, acute stroke imaging, pediatric neurological disorders, and cancers of the breast, bladder, and cervix, which demonstrates its versatility as a molecular MRI technique [13–18]. Recent studies have extended APT imaging to neurodegenerative diseases, showing its utility in detecting Alzheimer’s disease- and Parkinson’s disease-related brain changes [19, 20]. However, APT investigations of cerebral metabolism in T2DM patients remain very rare.

Given its sensitivity to molecular-level tissue changes, APT MRI has the potential to serve as a noninvasive biomarker for identifying cerebral metabolic alterations associated with T2DM-related cognitive decline, enabling timely intervention before irreversible damage occurs. We hypothesized that APT MRI could quantify such alterations in T2DM patients, and that APT values would correlate with both cognitive performance and CSVD imaging markers, potentially serving as a sensitive biomarker for early cognitive impairment in diabetes. This study aimed to test this hypothesis by comparing APT signals among T2DM patients with and without mild cognitive impairment together with healthy controls, and by examining the relationships between APT values and cognitive test scores or CSVD imaging markers.

## Methods

### Participants

This study was approved by the Ethics Committee of the First Affiliated Hospital of Dalian Medical University (Ethical Approval Ref: YJ-KY-FB-2020-08). T2DM patients and healthy controls were prospectively recruited. Inclusion criteria for the T2DM patient group were: (1) diagnosis of T2DM according to the 2014 American Diabetes Association diagnostic criteria [21]; (2) right-handed; (3) no contraindications to MRI (confirmed by MRI safety screening questionnaire); and (4) no significant brain abnormalities on conventional MRI aside from mild age-related changes such as minimal white matter hyperintensities. Exclusion criteria for the T2DM group included: presence of severe systemic medical conditions (e.g., moderate-to-severe anemia, organ failure, severe electrolyte imbalance), history of alcohol or drug abuse, any current or past neurological/psychiatric disorder or family history of such, history of major cerebrovascular events, major systemic diseases (e.g., malignancy, autoimmune disease, thyroid dysfunction), history of brain surgery, or poor quality MRI scans that precluded analysis.

Healthy control participants were matched to the T2DM group by age, sex, and education. Inclusion criteria for the control group required: (1) in good general health, no cognitive complaints or other significant medical conditions; and (2) right-handedness, MRI compatibility confirmed by safety screening, and normal brain MRI aside from mild age-related changes. Participants were excluded if MRI data were of suboptimal quality.

Based on diagnostic criteria for MCI, T2DM patients were subdivided into two groups: those with mild cognitive impairment (T2DM-MCI) and those with normal cognition (T2DM-NC). The diagnostic criteria for MCI are as follows [22]: (1) cognitive impairment reported by the chief complaint or informed person (such as a relative) ; (2) basic activities of daily living are normal, and complex instrumental activities of daily living may be slightly impaired. (3) did not meet the diagnostic criteria for dementia (4) MoCA score of 18–25 [23] (one point was added to the total score for participants with ≤ 12 years of education to correct for the influence of educational level). Cognitively normal controls were required to meet the following criteria: (1) no subjective cognitive complaints; (2) basic and instrumental activities of daily living are intact; (3) MoCA score ≥ 26 (with one point added to the total score for participants with ≤ 12 years of education to correct for the influence of educational level). All participants provided written informed consent prior to enrollment.

## Clinical characteristics

Clinical and demographic data were collected for all participants within one week before or after the MRI scan. Key variables included age, sex, years of education, duration of T2DM and body mass index (BMI). Glycemic control indices, specifically glycosylated hemoglobin (HbA1c) and fasting plasma glucose (FG) levels, were also measured due to their relevance to diabetes severity.

## Neuropsychological tests

All subjects underwent neuropsychological assessments, including the Montreal Cognitive Assessment (MoCA), Mini-Mental State Examination (MMSE), and the Symbol Digit Modalities Test (SDMT). The MMSE evaluates cognitive status across seven domains, including orientation (time and place), immediate and delayed recall, attention and calculation, language, and visuospatial skills. The MoCA assesses eight domains, including attention and concentration, executive function, memory, language, visuospatial skills, abstract reasoning, calculation, and orientation. The SDMT was used to assess cognitive abilities often affected early in diabetes, particularly processing speed and executive function. All evaluations were administered by two certified neuropsychologists who were blinded to participants’ group assignments to ensure unbiased scoring.

## Image acquisition

All participants were scanned using a 3.0 T Philips Ingenia CX MRI scanner (Philips Healthcare, Best, The Netherlands) equipped with a 32-channel head coil. Conventional brain MRI sequences were first acquired to screen for structural abnormalities, including axial T1-weighted imaging (T1WI), axial T2-weighted imaging (T2WI), axial T2-fluid attenuated inversion recovery (T2-FLAIR), and sagittal 3D T1WI (high-resolution T1). Detailed scan parameters for each sequence are provided in Table 1. APTw imaging was performed using an optimized 3D fast spin-echo sequence. Dual-source RF pulses with a B_1_ intensity (rms) of 2µT were applied to saturate each repeat acquisition for 2 s at specific frequency offsets. Data were acquired at seven saturation frequency offsets (± 2.7, ± 3.5, ± 4.3, and − 1540 ppm) to fit the z-spectra. B₀ maps were obtained at a saturation frequency of + 3.5 ppm using three different echo times for voxel-wise correction of the z-spectrum. The APT value was calculated as the asymmetry in the magnetization transfer ratio at ± 3.5 ppm after B_0_ correction, according to the following equation:

**Table 1:** MRI acquisition parameters

Sequence	TR (ms)	TE (ms)	TI (ms)	FOV (mm)	Matrix	NSA	Slices	Thickness (mm)	Flip Angle (°)
T1WI	297	6.9	–	230 × 183	232 × 178	1	50	3	75
T2WI	1186	13.8	–	230 × 183	256 × 164	1	50	3	90
T2-FLAIR	11,000	120	2800	230 × 184	240 × 143	1	50	3	90
3D T1WI	6.6	3	–	256 × 256	256 × 240	1	188	1	12
APT	5864	7.4	–	230 × 180	76 × 59	1	24	6	90

$$ \begin{aligned} {\mathrm{APTw}}\% {\text{ }} = & {\text{ MTR}}_{{{\mathrm{asym}}}} \left[ {{\mathrm{Dw}} = + 3.5\;{\mathrm{ppm}}} \right]{\text{ }} \\ & = {\text{ }}\left( {S_{{ - Dw}} - S_{{ + Dw}} } \right)/S_{0} \times 100\% , \\ \end{aligned} $$

where S_0_ is the water signal intensity at a saturation frequency of − 1540 ppm, and S_−Δω_ and S_+Δω_ are the water signal intensities with saturation at − 3.5 ppm and + 3.5 ppm, respectively. To optimize APT image quality, dynamic shimming was applied before each acquisition to improve local B₀ field homogeneity and reduce frequency drift during saturation. Dual RF transmission was used to enhance B₁ field homogeneity. Advanced fat- and fluid- suppression techniques provided by the Philips Ingenia CX system were also utilized to minimize artifacts from adipose tissue and cerebrospinal fluid, thereby enhancing APT contrast in brain tissues.

## Image analysis

**Fig. 1:**
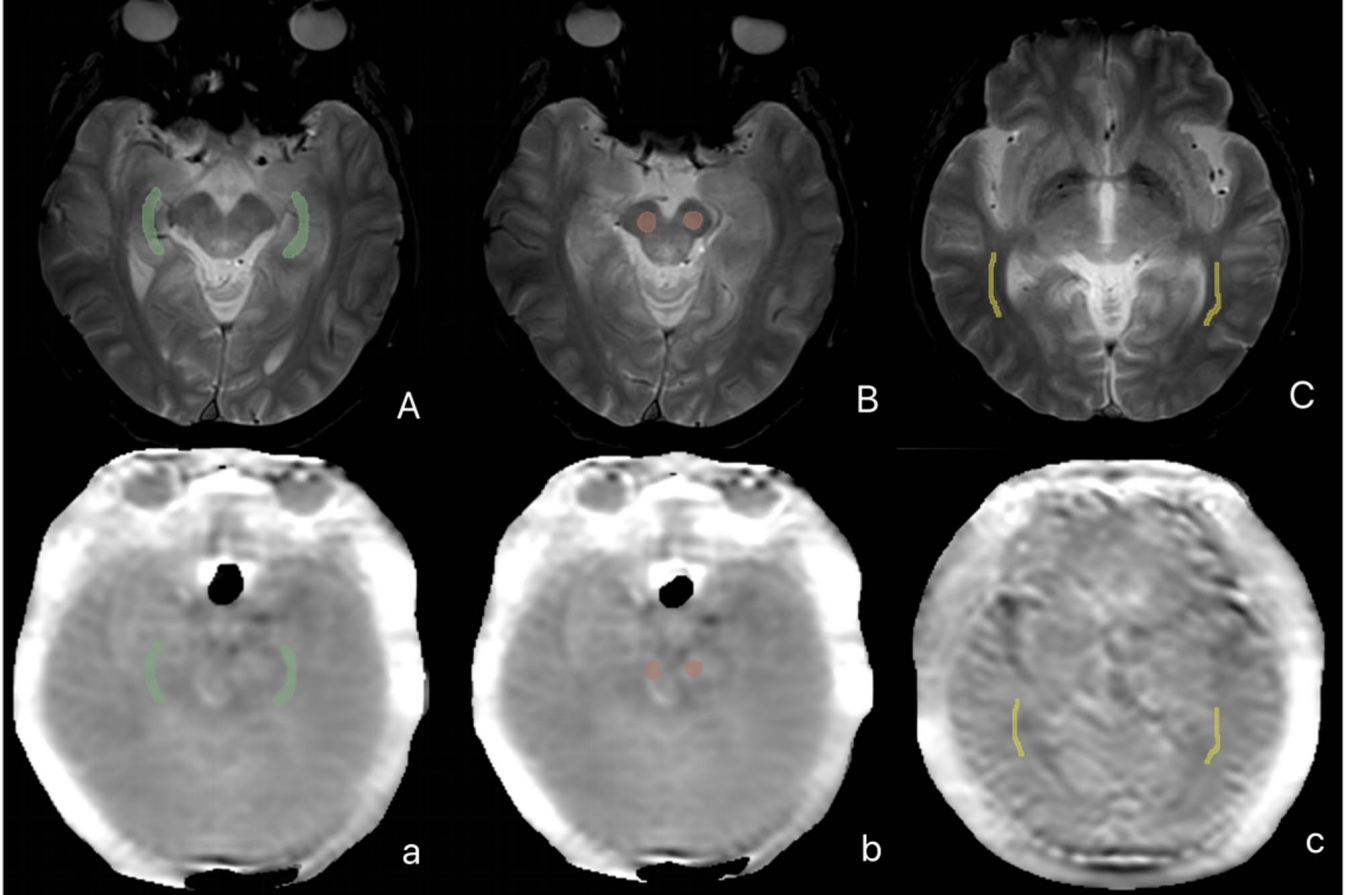
ROI placement examples on APT maps: bilateral hippocampi (Hipp), cerebral peduncles (CP), and temporal white matter (TWM)

**Fig. 2:**
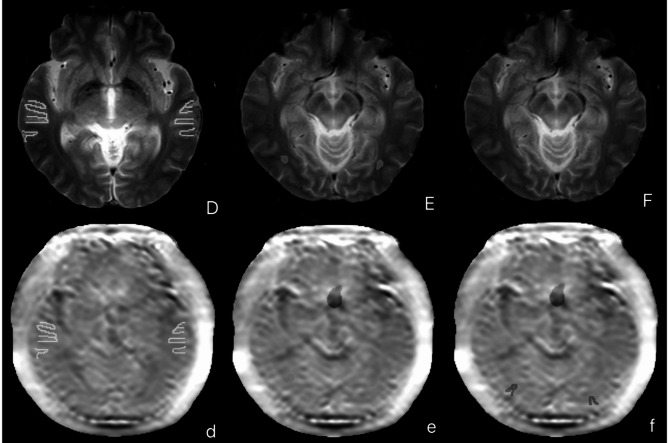
ROI placement examples on APT maps: temporal gray matter (TGM), occipital white matter (OWM), and occipital gray matter (OGM)

Quantitative analysis of APT maps was performed using 3D Slicer (v4.11.20210226). Regions of interest (ROIs) were manually delineated on the APT images with reference to co-registered T2-weighted images (T2WI) for anatomical guidance. Bilateral ROIs were manually placed in the hippocampus (Hipp), cerebral peduncle (CP), temporal lobe white matter (TWM), temporal lobe gray matter (TGM), occipital lobe white matter (OWM), and occipital lobe gray matter (OGM), as illustrated in Figs. 1 and 2. Care was taken to carefully avoid adjacent cerebrospinal fluid spaces to minimize partial volume effects. For each ROI, the mean APT signal intensity was calculated. ROIs were delineated independently by two neuroradiologists (each with more than 5 years of experience) blinded to clinical data. Inter-rater consistency was assessed by calculating the intraclass correlation coefficient (ICC). The final results were based on the measurements from the more experienced neuroradiologist.

**Fig. 3:**
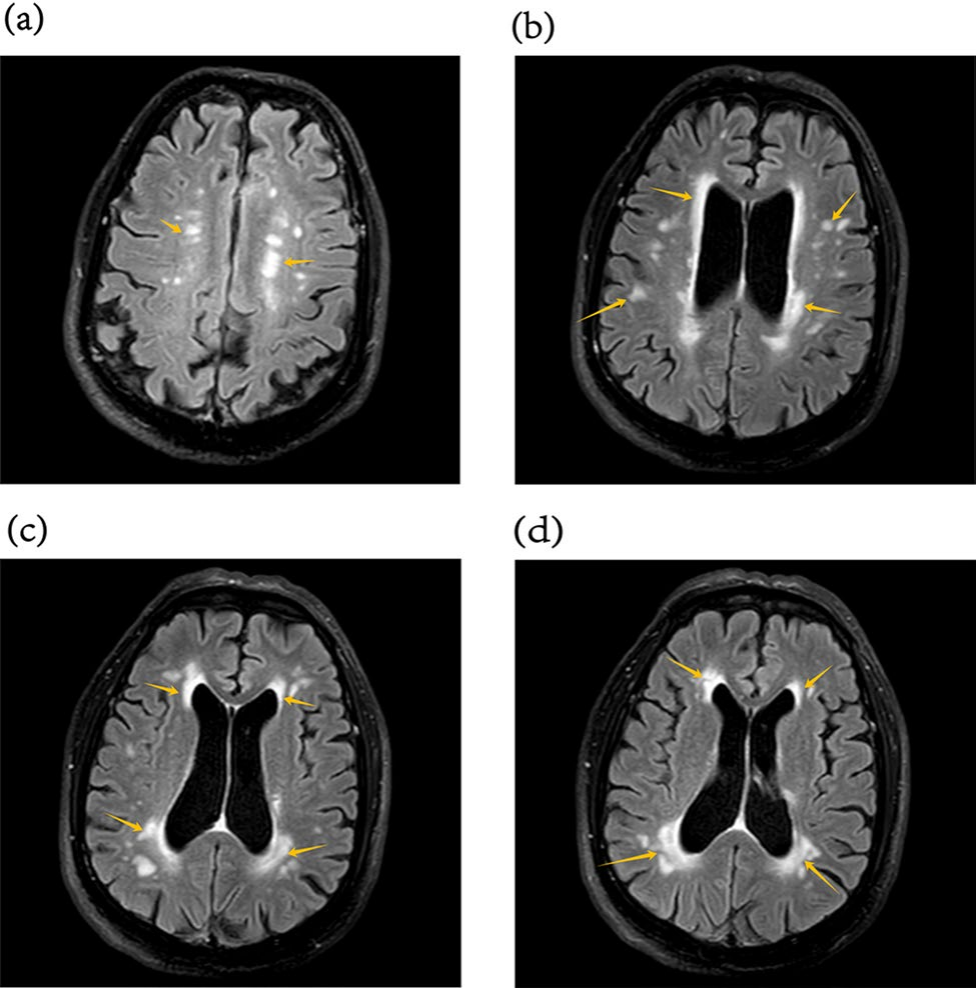
Spatial distribution of periventricular white matter hyperintensities (WMH) in a patient with T2DM-MCI

Imaging markers of CSVD were automatically identified and quantified using an AI-assisted image analysis tool (United Imaging, Shanghai, China) applied to the conventional MRI sequences. This tool detected and measured white matter hyperintensities (WMH), lacunar infarctions (LI), cerebral microbleeds (CMB), and basal ganglia enlarged perivascular spaces (EPVS) in accordance with the STRIVE-2 neuroimaging standards for reporting vascular changes [24]. A CSVD burden score (ranging from 0 to 4) was calculated for each subject by summing the presence of each of the four markers. Figure 3 illustrates a typical periventricular WMH pattern of CSVD in our cohort.

### Statistical analysis

Statistical analyses were performed using SPSS Statistics (v27.0, IBM Corp, Armonk, NY, USA). Data normality was assessed using the Shapiro–Wilk test. For between-group comparisons, one-way ANOVA was applied for normally distributed continuous variables, followed by Bonferroni-corrected post hoc tests for pairwise comparisons. For non-normally distributed variables, the Kruskal–Wallis test was used with Bonferroni-corrected post hoc comparisons (Nemenyi’s test). Categorical variables were compared using chi-square tests; if the chi-square was significant, pairwise comparisons with Bonferroni correction were conducted. Within the T2DM group, partial correlation analyses (Bonferroni-corrected) were performed to examine associations between regional APT values and clinical or cognitive measures (MoCA, MMSE, SDMT) as well as CSVD scores, controlling for age, sex, education level and duration of T2DM. A two-tailed *P* < 0.05 was considered statistically significant, after Bonferroni correction where applicable.

## Results

### Participant characteristics

A total of 58 subjects were enrolled: 16 patients in the T2DM-MCI group, 16 in the T2DM-NC group, and 26 healthy controls (HC). No significant differences were found among the three groups in age, sex, or years of education (all *P* > 0.05). The T2DM-MCI group had significantly lower MoCA score and MMSE score than the T2DM-NC group (MoCA score mean 23.69 ± 1.25 vs. 27.44 ± 1.41, *P* < 0.001 and MMSE score mean 27.69 ± 1.45 vs. 28.69 ± 1.01, *P* = 0.019). The two T2DM groups had similar diabetes duration and glycemic indices. No significant inter-group differences were observed in CSVD burden score or any individual MRI-visible CSVD markers (WMH, LI, CMB, EPVS) (all *P* > 0.05 in overall comparisons). Detailed results are shown in Table 2 (MMSE and MoCA subscale scores of the participants are shown in Supplementary material-Table 1).

**Table 2:** Demographics, Cognitive Scores, and CSVD Markers of the participants

Variables	T2DM-MCI (n = 16)	T2DM-NC (n = 16)	HC (n = 26)	*P*
Demographics				
Age(yr),	63.13 ± 5.11	59.56 ± 8.12	59.19 ± 6.18	*0.106*
Male, n(%)	7(44)	9(56)	8(31)	*0.259*
Education(yr)	10.00 ± 2.53	12.44 ± 2.87	12.27 ± 3.56	*0.054*
Duration of T2DM(yr)	6.34 ± 4.59	11.31 ± 9.51	-	*0.069*
BMI (kg/m^2^)	25.30 ± 4.00	25.60 ± 2.80	23.50 ± 3.30	*0.081*
MMSE	27.69 ± 1.45	28.69 ± 1.01	28.85 ± 0.92	*0.019*
MoCA	23.69 ± 1.25	27.44 ± 1.41	26.19 ± 2.51	< *0.001*
SDMT	30.44 ± 14.00	39.69 ± 15.01	41.08 ± 14.41	*0.061*
Laboratory data				
HbA1c (%)	7.33 ± 1.38	7.72 ± 1.56	5.52 ± 0.28	< *0.001*
FG (mmol/L)	7.17 ± 2.28	8.65 ± 2.53	4.87 ± 0.47	< *0.001*
Imaging indicators				
CSVD total burden	0.63 ± 0.89	0.75 ± 0.77	0.73 ± 0.60	*0.629*
Lacunar Infarcts (count)	0.44 ± 0.63	0.56 ± 1.03	0.42 ± 0.81	*0.812*
WMH	1.06 ± 0.25	1.06 ± 0.25	1.15 ± 0.37	*0.530*
CMB	0.38 ± 1.09	0.50 ± 0.90	0.31 ± 0.55	*0.536*
EPVS	0.69 ± 0.60	0.63 ± 0.50	0.69 ± 0.62	0.967

BMI: Body Mass Index, MoCA: Montreal cognitive assessment, MMSE: Mini-mental state examination, SDMT: Symbol Digit Modalities test, CSVD: Cerebral small vessel disease, WMH: white matter hyperintensities, CMB: cerebral microbleeds, BG-EPVS: basal ganglia-enlarged perivascular spaces

### Intergroup comparison of APT values

**Fig. 4:**
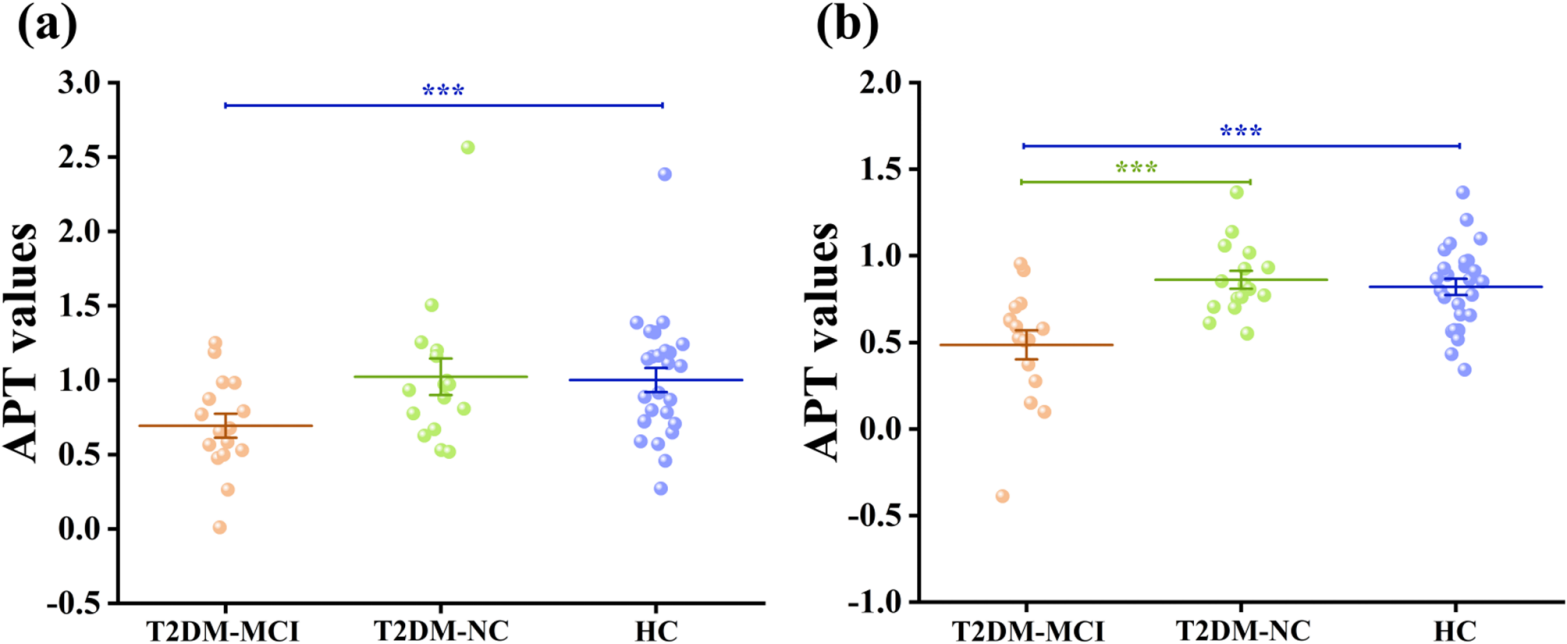
**a** Group comparison of right temporal lobe gray matter (TGM) APT values; **b** Group comparison of left temporal lobe white matter (TWM) APT values. *** represents a significant difference. In **a**, *P* = 0.044; in **b**, green indicates *P* = 0.002 and blue indicates *P* = 0.003

**Fig. 5:**
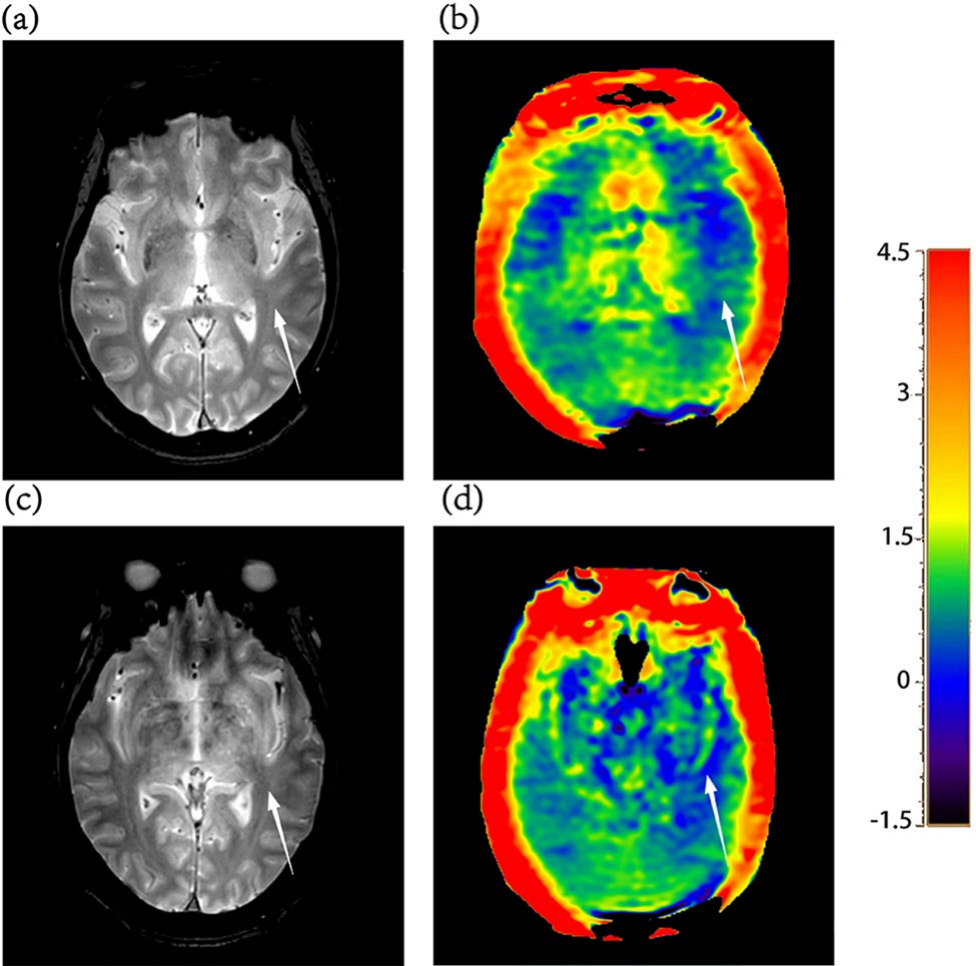
**a** T2-weighted image and **b** APT-weighted image of a healthy control (male, 61 years old, MoCA score 26). **c** T2-weighted image and (d) APT-weighted image of a T2DM-MCI patient (female, 68 years old, MoCA score 23). The APT-weighted signal (white arrow) in the left TWM region was lower in the T2DM-MCI patient than in the healthy control

The ICC values ranged from 0.680 to 0.910 (*P* < 0.050), indicating good inter-rater agreement between the two observers. APT values were compared across the three groups for each ROI (Table 3). Figure 4 illustrates the regions with significant group differences. In the left TWM, APT values differed significantly among the groups (*P* < 0.001, T2DM-MCI group’s APT values = 0.50 ± 0.30, T2DM-NC group’s APT values = 0.82 ± 0.22, HC group’s APT values = 0.82 ± 0.23). Post hoc analysis showed that the T2DM-MCI group had significantly lower left TWM APT value than both the T2DM-NC group (Bonferroni-corrected *P* = 0.002) and healthy controls (Bonferroni-corrected *P* = 0.003), whereas the T2DM-NC group did not differ significantly from controls. In the right TGM, there was a significant overall group effect (*P* = 0.035, T2DM-MCI group’s APT values = 0.69 ± 0.40, T2DM-NC group’s APT values = 1.04 ± 0.44, HC group’s APT values = 1.00 ± 0.41). The T2DM-MCI group had significantly lower right TGM APT values compared with healthy controls (Bonferroni-corrected *P* = 0.044), while the T2DM-NC group did not differ significantly from either group. No significant differences were observed in the hippocampus, occipital lobe, or cerebral peduncle ROIs (all *P* > 0.05 Table 3). Figure 5 presents representative T2WI and APTw images from a healthy control and a T2DM-MCI patient, demonstrating reduced TWM APT values in the patient. Potential outliers were identified using the interquartile range (IQR) method. Sensitivity analyses, performed by either excluding outliers or replacing them with group means, yielded results consistent with the primary findings for the left TWM. For the right TGM, the difference remained significant after mean replacement, whereas exclusion of outliers rendered the result marginally non-significant after Bonferroni correction, but the overall trend remained (see Supplementary Materials).

**Table 3:** Group comparison of APT values (% signal)

ROI	T2DM-MCI	T2DM-NC	HC	*P*
Right Hipp	1.18 ± 0.60	1.01 ± 0.28	1.17 ± 0.41	*0.801*
Left Hipp	1.03 ± 0.45	1.02 ± 0.25	1.02 ± 0.41	*0.668*
Right CP	0.83 ± 0.98	0.90 ± 0.43	1.00 ± 0.66	*0.255*
Left CP	0.66 ± 0.65	0.95 ± 0.50	1.10 ± 0.83	*0.116*
Right TWM	0.85 ± 0.40	0.96 ± 0.28	0.91 ± 0.43	*0.293*
Left TWM	0.50 ± 0.30	0.82 ± 0.22	0.82 ± 0.23	<0.001*
Right TGM	0.69 ± 0.40	1.04 ± 0.44	1.00 ± 0.41	0.035*
Left TGM	0.78 ± 0.41	0.76 ± 0.43	0.74 ± 0.42	*0.878*
Right OWM	0.88 ± 0.26	0.97 ± 0.25	0.97 ± 0.33	*0.717*
Left OWM	0.88 ± 0.32	0.88 ± 0.19	0.95 ± 0.41	*0.421*
Right OGM	0.97 ± 0.37	1.06 ± 0.20	1.03 ± 0.35	*0.936*
Left OGM	0.99 ± 0.25	1.18 ± 0.24	1.19 ± 0.52	*0.200*

*Significant change. Hipp: Hippocampus, CP: Cerebral peduncles, TWM: Temporal white matter, TGM: Temporal gray matter, OWM: Occipital white matter, OGM: Occipital gray matter. *P* values were corrected by Bonferroni for multiple comparisons

### Correlations between APT values and clinical variables

**Fig. 6:**
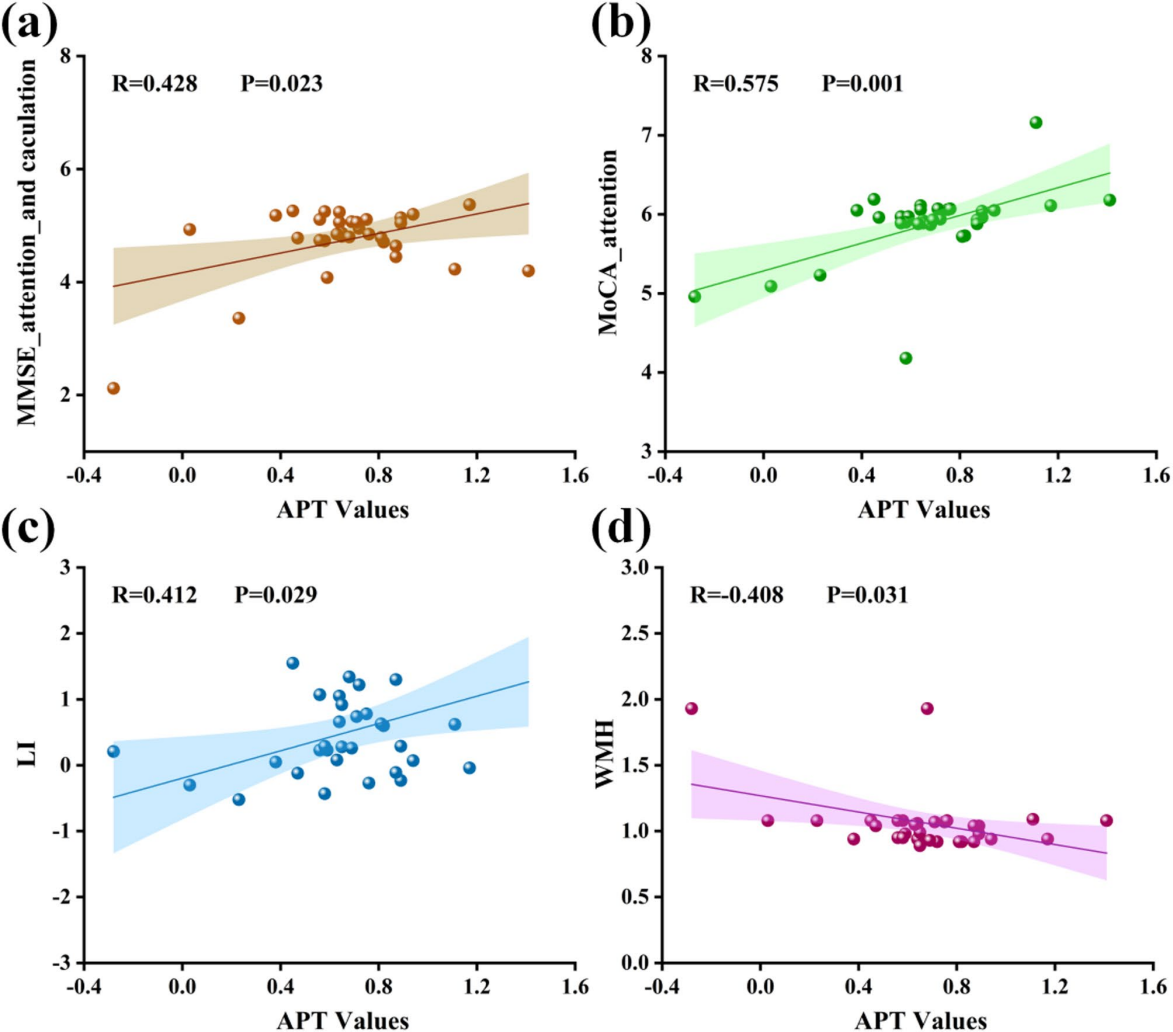
Scatter plot of partial correlation for T2DM cohort: **a** left TWM APT vs. MMSE attention and calculation score (*r* = 0.428, *P* = 0.023); **b** left TWM APT vs. MoCA attention score (*r* = 0.575, *P* = 0.001); **c** left TWM APT vs. the number of LI (*r* = 0.412, *P* = 0.029); **d** left TWM APT vs. WMH score (*r* = -0.408, *P* = 0.031). All P values were Bonferroni-corrected

**Fig. 7:**
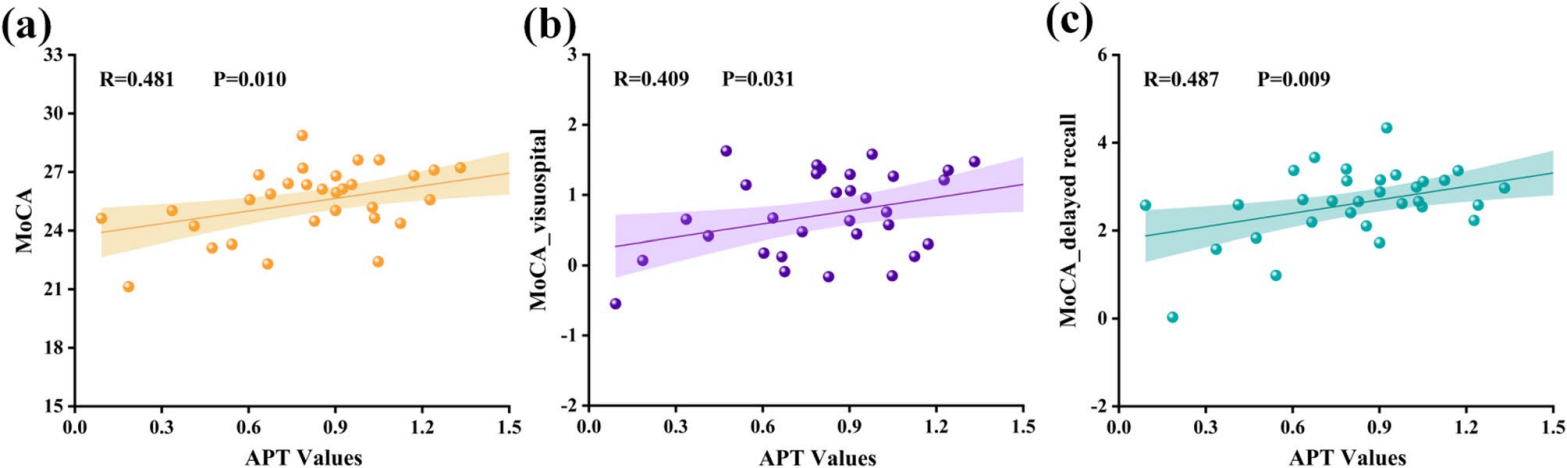
Scatter plot of partial correlation in T2DM cohort: **a** right TGM APT vs. MoCA total score (*r* = 0.458, *P* = 0.014); **b** Right TGM APT vs. MoCA delayed recall score (*r* = 0.461, *P* = 0.014); **c** Right TGM APT vs. EPVS rating (*r* = 0.385, *P* = 0.043). All P values were Bonferroni-corrected

Within the combined T2DM patient cohort (*n* = 32), partial correlation analyses were performed between APT values and cognitive test scores or CSVD imaging markers, controlling for age, sex, education level, and duration of T2DM. APT values in the left TWM showed a moderate positive correlation with MMSE attention and calculation score (*r* = 0.428, Bonferroni-corrected *P* = 0.023), and the MoCA attention score (*r* = 0.575, Bonferroni-corrected *P* = 0.001). But no significant correlation with total MMSE, total MoCA, or SDMT scores. In addition, left TWM APT values were positively correlated with the number of lacunar infarctions (*r* = 0.412, Bonferroni-corrected *P* = 0.029) and negatively correlated with WMH scores (*r* =–0.408, Bonferroni-corrected *P* = 0.031), but showed no significant association with the overall CSVD burden score. In contrast, right TGM APT values were significantly correlated with the MoCA total score (*r* = 0.481, Bonferroni-corrected *P* = 0.010) and with the MoCA visuospatial (*r* = 0.409, Bonferroni-corrected *P* = 0.031) and delayed recall (*r* = 0.487, Bonferroni-corrected *P* = 0.009) scores. No significant correlations were found with SDMT, total MMSE score, MMSE subscores, or CSVD imaging markers. No significant associations were observed between APT values in the hippocampus, occipital lobe, or cerebral peduncles and either cognitive scores or CSVD imaging markers (all *P* > 0.05). Scatter plots of correlations are presented in Figs. 6 and 7.

## Discussion

In this study, we employed APT-weighted MRI to assess cerebral metabolic alterations in T2DM and their associations with cognitive performance and CSVD imaging markers. APT values in the temporal lobes were significantly lower in T2DM-MCI than in both T2DM-NC and healthy controls. Within the T2DM cohort, regional APT values correlated significantly with MoCA and MMSE scores, as well as with CSVD markers. These findings indicate that APT MRI can detect subtle metabolic alterations linked to early cognitive impairment in T2DM, supporting its potential as a non-invasive biomarker for diabetic cognitive dysfunction.

APT-weighted contrast is primarily determined by the concentration and exchange rate of mobile amide protons, modulated by pH and temperature, and further influenced by water T_1_ relaxation, the nuclear Overhauser effect (NOE), and tissue water content [12]. In this study, temporal lobe APT values were reduced in T2DM patients with MCI, likely reflecting combined effects of impaired insulin signaling, chronic hyperglycemia–induced accumulation of advanced glycation end-products (AGEs), oxidative stress, and cerebral microangiopathy [25, 26]. In addition, T2DM brains may also present Alzheimer’s disease (AD)-like pathology such as Aβ deposition or tau abnormalities [27, 28]. Hyperglycemia-driven AGE formation promotes protein cross-linking and insolubilization, thereby reducing mobile protein/peptide pools and altering the CEST environment. This process ultimately lowers APT contrast [25]. Oxidative stress can cause protein oxidation, reduce 26 S proteasome activity, and impair autophagy, allowing oxidatively modified proteins to accumulate in cells. Initially, these oxidized proteins may remain in a free state, but over time they tend to aggregate; in cases of severe oxidative damage, cross-linking and aggregation occur, leading to a subsequent decline in free protein levels [29]. Microvascular injury induces hypoperfusion and metabolic dysfunction, resulting in tissue acidification that slows amide proton exchange and lowers APT values. A recent neuroimaging study indicated that in T2DM patients, white matter microstructural damage is characterized by reduced axonal density, increased fiber dispersion, and vasogenic edema secondary to blood–brain barrier disruption [30]. Such changes may increase tissue hydration and further dilute the APT contrast. AD–like pathology may alter protein homeostasis by increasing the pool of mobile proteins and peptides, potentially elevating APT values. Overall, the net effect of these diverse pathological processes on APT values may be bidirectional—either increasing or decreasing—depending on the disease stage and tissue condition.The decrease in temporal lobe APT observed in T2DM-related cognitive impairment in our study may reflect a composite dysregulation of protein metabolism and homeostasis. To date, peer-reviewed studies on APT changes in the brains of T2DM patients remain scarce. Our results provide preliminary evidence that APT could serve as a molecular imaging biomarker of metabolic changes in the brains of T2DM-MCI patients, and its effectiveness awaits validation in larger longitudinal cohorts.

In contrast to our results, Zhang et al. reported increased APT values in multiple brain regions of patients with amnestic mild cognitive impairment (aMCI) [31]. As the prodromal stage of the Alzheimer’s disease (AD) continuum, aMCI is characterized by abnormal deposition of β-amyloid plaques and tau neurofibrillary tangles [32], which may involve pathological processes distinct from those in T2DM-related MCI. Notably, previous human studies in aMCI/AD consistently report elevated APT values [31, 33, 34], whereas AD animal models often demonstrate decreased values [35]. Divergent findings across human and animal studies, as well as in other conditions such as demyelinating diseases [36], suggest that APT alterations are influenced by disease stage, pathology type, and quantification method.This underscores the need for pathology-correlated and longitudinal research.

The more marked APT reduction in T2DM-MCI versus T2DM-NC suggests additional pathology at the MCI stage, in line with evidence that diabetic MCI patients show more severe structural brain alterations than cognitively normal T2DM [37, 38]. This suggests that metabolic changes detectable by APT may only become apparent when cognitive impairment occurs, indirectly demonstrating the potential of APT for detecting brain metabolic changes in T2DM patients with MCI. The temporal lobe, especially the medial region, is among the earliest affected in T2DM-related cognitive decline [39, 40]. This may explain why APT reductions were localized to this area.

We observed left-lateralized APT reductions, most prominent in left temporal white matter. Prior studies show greater left-hemisphere vulnerability in T2DM, with more pronounced cortical loss [41], predominant FA reductions in left temporal white matter linked to verbal memory impairment [42], and similar findings in T2DM-MCI [43]. The precise mechanism underlying this asymmetry remains to be further explored, and studies using whole-brain voxel-based analysis with pathological correlation are needed to confirm this lateralization.

In our T2DM cohort, temporal lobe APT values were associated with cognitive performance, particularly attention and delayed recall, consistent with the temporal lobe’s role in these domains. As APT reflects tissue metabolism, its reduction in T2DM-MCI likely indicates metabolic abnormalities that may contribute to cognitive decline. Similar associations have been reported in AD and aMCI, including correlations between hippocampal MTR_asym and MMSE in AD, reduced APT linked to cognitive deficits in an AD rat model, and APT values in aMCI related to and predictive of cognitive status [19, 34, 35, 44]. Notably, no correlation was found with SDMT. This test primarily assesses processing speed and executive function, and these abilities are more dependent on frontoparietal network activity [45, 46]. These findings suggest that APT–cognition associations may be brain region– and domain-specific.

APT also correlated with CSVD markers consistent with prior findings that APT reflects WMH pathophysiology [47]. A study showed in non-diabetic populations, hippocampal APT values were negatively associated with CSVD burden [48], supporting APT as a biomarker of microvascular-related metabolic injury. However, this result in our study is unstable (after dealing with outliers) and requires validation with a larger sample.

This study has several limitations. First, ROIs were manually delineated, which is inherently subjective and may introduce bias; future studies could employ automated or atlas-based ROI methods to improve objectivity and reproducibility. Second, the neuropsychological assessment was limited to the MMSE, MoCA, and SDMT, and future research should incorporate more domain-specific tests to strengthen correlations with APT signal changes. Third, the relatively small sample size limits the statistical power to detect subtle associations, especially when analyzing multiple variables. Multi-center studies with larger cohorts are needed to validate these findings and enhance generalizability. Fourth, while our cross-sectional data link APT values to cognitive status, longitudinal research is essential to determine whether baseline APT metrics can predict future cognitive decline. Although no longitudinal APT studies in T2DM have been reported, dynamic APT changes have been observed in other conditions such as ischemic stroke [49]. We are currently conducting follow-up assessments to address this question. Fifth, the deliberate exclusion of comorbidities further limits the generalizability of our results. Future studies should include patients with controlled comorbidities to more accurately reflect the characteristics of the real-world population.

## Conclusions

APT-weighted MRI detected temporal lobe metabolic alterations in T2DM-MCI, correlating with both cognitive performance and CSVD markers. These findings offer insight into the metabolic mechanisms of diabetic cognitive impairment and support APT as a non-invasive biomarker for early detection in T2DM.

## Supplementary Information

Below is the link to the electronic supplementary material.

Supplementary Material 1

## Data Availability

The datasets used and/or analysed during the current study are available from the corresponding author on reasonable request.

## Abbreviations

APT: Amide proton transfer
T2DM: Type 2 diabetes mellitus
MCI: Mild cognitive impairment
NC: Normal cognition
CSVD: Cerebral small vessel disease
TWM: Temporal white matter
TGM: Temporal gray matter
MoCA: Montreal Cognitive Assessment
MMSE: Mini-Mental State Examination
CEST: Chemical exchange saturation transfer
BMI: Body mass index
FG: Fasting plasma glucose
SDMT: Symbol Digit Modalities Test
Hipp: Hippocampus
CP: Cerebral peduncle
OWM: Occipital lobe white matter
OGM: Occipital lobe gray matter
WMH: White matter hyperintensities
LI: Lacunar infarctions
CMB: Cerebral microbleeds
EPVS: Enlarged perivascular spaces
