# Supplementary material for: Amide proton transfer imaging reveals cerebral metabolic alterations associated with cognitive impairment in type 2 diabetes mellitus
Source: J Transl Med. 2025 Oct 7;23:1059. doi: 10.1186/s12967-025-07083-0 (PMC12502362; doi:10.1186/s12967-025-07083-0)
Supplement: Supplementary file 1 — Supplementary Material 1 [file 12967_2025_7083_MOESM1_ESM.docx]

**Supplementary Sensitivity Analysis for Potential Outliers**

**Method**

To assess the robustness of the results, potential outliers were formally identified using the interquartile range (IQR) method. The first quartile (Q1) and third quartile (Q3) were calculated, and the IQR was obtained by subtracting Q1 from Q3. Data points lying outside the range (Q1 – 1.5 × IQR, Q3 + 1.5 × IQR) were considered outliers. Based on this criterion, we identified one outlier in the left temporal white matter and two in the right temporal gray matter. Two sensitivity analyses were then performed: (1) exclusion of identified outliers, and (2) replacement of outliers with the corresponding group mean values.

**Results**

**Intergroup Comparison of APT Values**

1. The results of excluding outlier APT values: A significant difference was found in APT values of left temporal white matter among the three groups (*P* = 0.002). Post-hoc pairwise comparisons with Bonferroni correction revealed that the T2DM-MCI group had significantly lower APT values compared to both the T2DM-NC group (*P* = 0.004) and the HC group (*P* = 0.005). However, no significant difference was detected between the T2DM-NC and HC groups. For right temporal gray matter APT values, no significant intergroup differences were observed (*P* = 0.053).
2. The results of mean replacement of outlier APT values: A significant difference was found in APT values of left temporal white matter among the three groups (*P* < 0.001). Post-hoc analysis with Bonferroni correction indicated lower APT values in the T2DM-MCI group relative to the T2DM-NC group (*P* = 0.003) and HC group (*P* = 0.003), while the T2DM-NC and HC groups remained comparable. Right temporal gray matter APT values showed nominal intergroup differences (*P* = 0.048), but no pairwise differences reached significance after Bonferroni correction (T2DM-MCI vs T2DM-NC *P* = 0.170, T2DM-MCI vs HC *P* = 0.055, T2DM-NC vs HC *P* = 1.000). Figure 1 shows the intergroup comparison of APT Values.

**Correlations Between APT Values and Clinical Variables**

1. After excluding the outliers, the APT value of the left temporal white matter was positively correlated with the MoCA total score (r = 0.387, Bonferroni-corrected *P* = 0.046), MoCA attention score (r = 0.481, Bonferroni-corrected *P* = 0.011), and the number of lacunar infarctions (r = 0.455, Bonferroni-corrected *P* = 0.017). However, no significant correlations were found with the SDMT, MMSE, or total CSVD burden scores. APT values in the right temporal gray matter were positively correlated with the MoCA total score (r = 0.461, Bonferroni-corrected *P* = 0.015), MoCA delayed recall score (r = 0.464, Bonferroni-corrected *P* = 0.015), and EPVS (r = 0.383, Bonferroni-corrected *P* = 0.049). However, no significant correlations were found with SDMT, MMSE, or other cerebral small vessel disease markers.
2. When the outliers in the left temporal white matter were replaced by the mean value, the APT value of the left temporal white matter was positively correlated with the MoCA total score (r = 0.381, Bonferroni-corrected *P* = 0.046), MoCA attention score (r = 0.474, Bonferroni-corrected *P* = 0.011), and the number of lacunar infarctions (r = 0.458, Bonferroni-corrected *P* = 0.014). However, no significant correlations were found with the SDMT, MMSE, or total CSVD burden scores. APT values in the right temporal gray matter remained positively correlated with the MoCA total score (r = 0.451, Bonferroni-corrected *P* = 0.016), MoCA delayed recall score (r = 0.453, Bonferroni-corrected *P* = 0.015), and EPVS (r = 0.381, Bonferroni-corrected *P* = 0.046). However, no significant correlations were observed with SDMT, MMSE, or other cerebral small vessel disease markers.

Correlation analysis between the APT values of the left temporal white matter and MoCA total score, MoCA attention score, and the number of lacunar infarctions are shown in Figure 2.

**Conclusions**

Following both the exclusion and mean-replacement, the intergroup difference results for left temporal white matter APT values were consistent with the original analysis, indicating stability. Using either the exclusion or mean-replacement method, the intergroup difference in right temporal gray matter APT values became marginally non-significant after Bonferroni correction (*P* slightly above 0.05), but the trend remained consistent with the initial analysis.

After both exclusion and mean-replacement, the correlations between left temporal white matter APT values and MoCA attention score and lacunar infarctions, as well as between right temporal gray matter APT values and MoCA total score and MoCA delayed recall score, remained consistent with the initial analysis, indicating stability, whereas all other correlations were unstable.


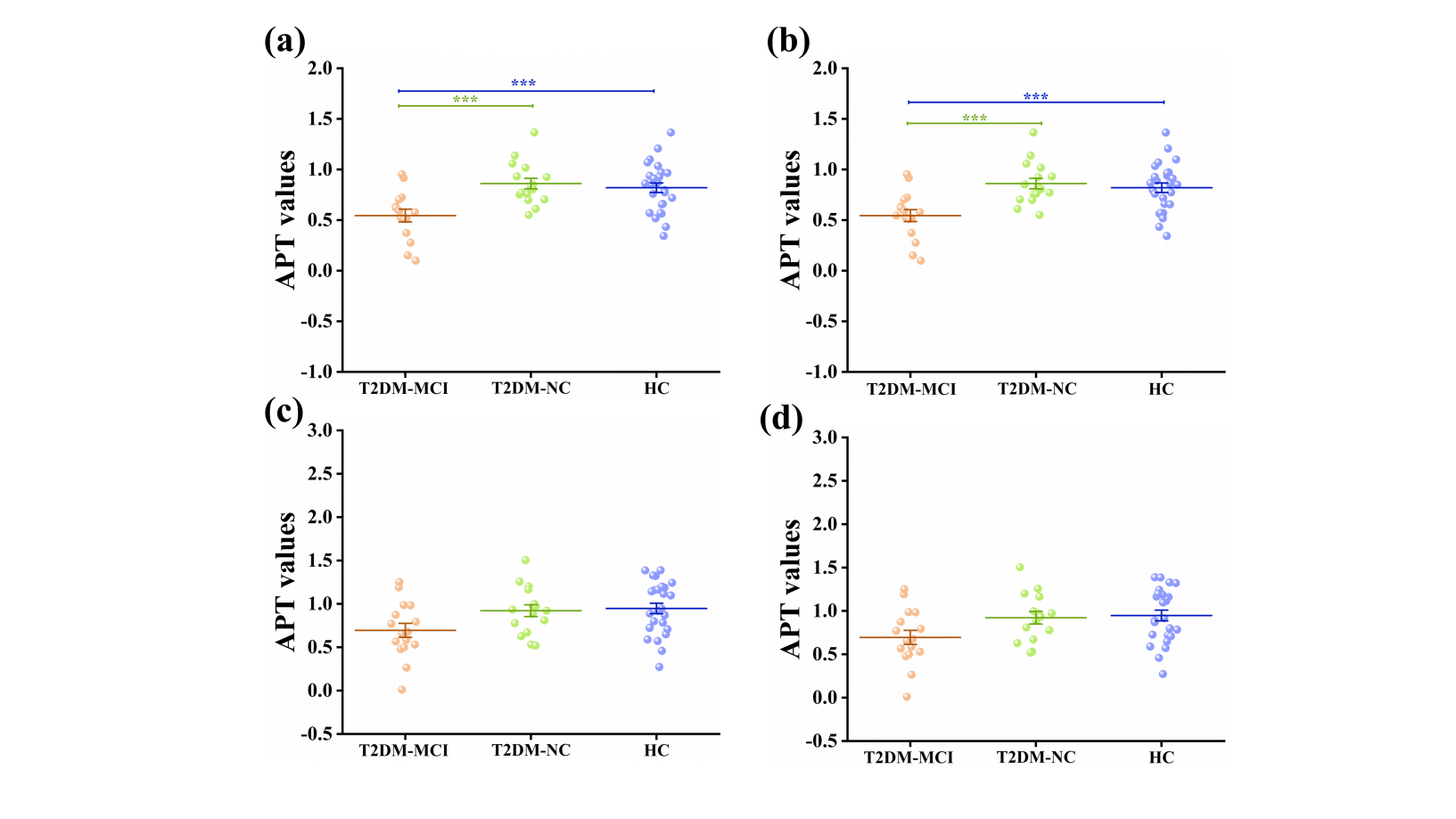


Figure 1 (a) Significant difference was found in APT values of the left temporal white matter among the three groups after excluding outliers. (b) Significant difference was found in APT values of the left temporal white matter among the three groups after mean replacement of outliers. (c) No significant difference was found in APT values of the right temporal gray matter among the three groups after excluding outliers. (d) No significant difference was found in APT values of the right temporal gray matter among the three groups after mean replacement of outliers. *** represents a significant difference, (a) green represents *P* = 0.004, blue represents *P* = 0.005. (b) green represents *P* = 0.003, blue represents *P* = 0.003.


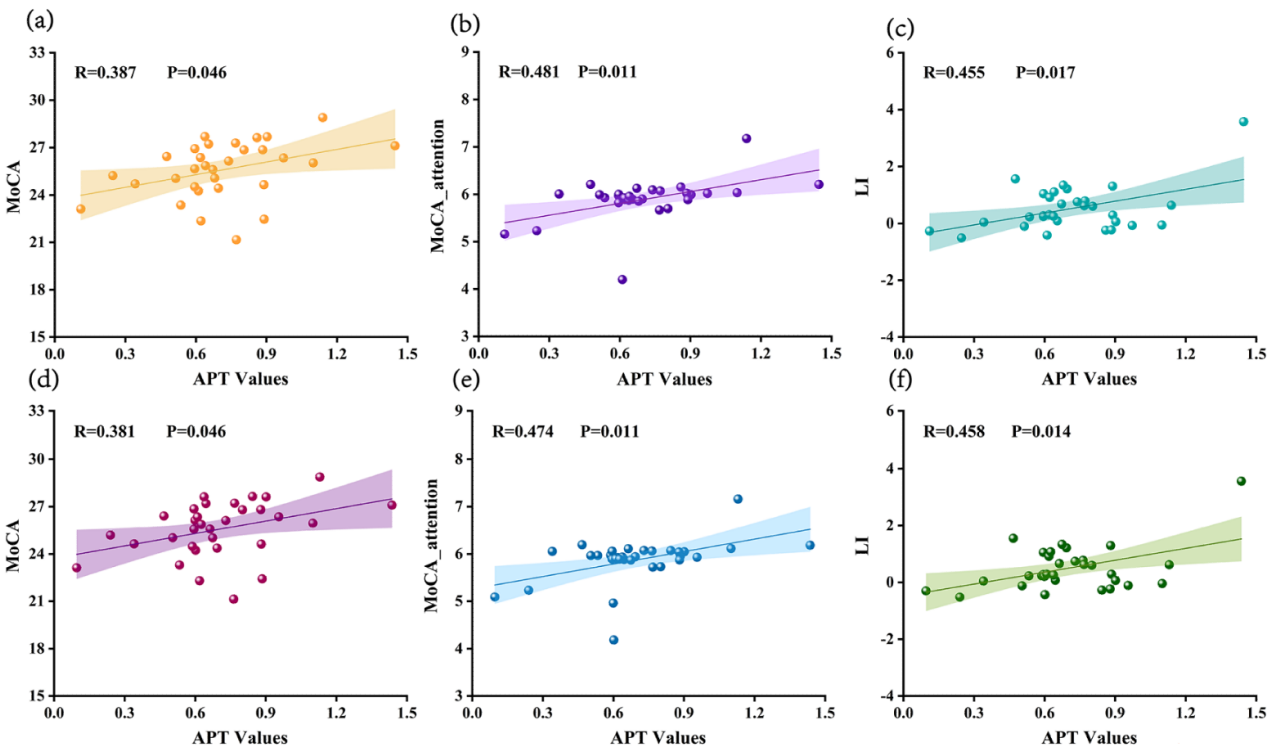


Figure 2 (a), (b), and (c) are scatter plots of the correlation between the APT values of the left temporal white matter and MoCA total score, MoCA attention score, and the number of lacunar infarctions after excluding the outliers in the APT value of the left temporal white matter (age, sex, education level and duration of T2DM as covariates). (d), (e), and (f) are scatter plots of the correlation between APT values of the left temporal white matter and MoCA total score, MoCA attention score, and the number of lacunar infarctions after replacing outlier values in APT value of the left temporal white matter with mean value (age, sex, education level and duration of T2DM as covariates).

**Table 1. MMSE and MoCA subscale scores of the participants**

| **Variables** | **T2DM-MCI**  **(n=16)** | **T2DM-NC**  **(n=16)** | | **HC**  **(n=26)** | | ***P*** |
| --- | --- | --- | --- | --- | --- | --- |
| MMSE | 27.69±1.45 | | 28.69±1.01 | | 28.85±0.92 | *0.019* |
| MMSE-orientation | 10.00±0.00 | | 10.00±0.00 | | 9.96±0.20 | *0.540* |
| MMSE-immediate recall | 3.00±0.00 | | 3.00±0.00 | | 3.00±0.00 | *1.000* |
| MMSE-attention and calculation | 4.63±0.89 | | 4.88±0.34 | | 4.88±0.43 | *0.535* |
| MMSE-delay recall | 1.50±1.1 | | 1.88±0.81 | | 2.19±0.94 | *0.093* |
| MMSE-language (naming) | 1.875±0.5 | | 2.00±0.00 | | 2.00±0.00 | *0.269* |
| MMSE-language (repetition) | 0.94±0.25 | | 1.00±0.00 | | 1.00±0.00 | *0.269* |
| MMSE-language (reading) | 1.00±0.00 | | 1.00±0.00 | | 1.00±0.00 | *1.000* |
| MMSE-language (command) | 3.00±0.00 | | 3.00±0.00 | | 3.00±0.00 | *1.000* |
| MMSE-language (writing) | 0.875±0.34 | | 1.00±0.00 | | 1.00±0.00 | *0.069* |
| MMSE-construction | 0.873±0.34 | | 0.94±0.25 | | 0.81±0.40 | *0.494* |
| MoCA | 23.69±1.25 | | 27.44±1.41 | | 26.19±2.51 | *＜0.001* |
| MoCA-visuospatial | 0.25±0.58 | | 1.25±0.68 | | 1.15±0.88 | *＜0.001* |
| MoCA-draw clock | 2.25±0.86 | | 3.00±0.00 | | 2.54±0.95 | *0.003* |
| MoCA-naming | 3.00±0.00 | | 3.00±0.00 | | 3.00±0.00 | *1.000* |
| MoCA-attention | 5.69±0.60 | | 6.06±0.25 | | 5.77±0.51 | *0.057* |
| MoCA-language | 2.38±0.72 | | 3.00±0.00 | | 2.69±0.62 | *0.006* |
| MoCA-abstraction | 1.94±0.25 | | 2.00±0.00 | | 2.00±0.00 | *0.269* |
| MoCA-delayed recall | 2.19±0.91 | | 3.13±0.96 | | 2.92±1.26 | *0.032* |
| MoCA-orientation | 6.00±0.00 | | 6.00±0.00 | | 6.00±0.00 | *1.000* |
